# What’s Worrying Our Students? Increasing Worry Levels over Two Decades and a New Measure of Student Worry Frequency and Domains

**DOI:** 10.1007/s10608-021-10270-0

**Published:** 2021-10-09

**Authors:** Graham C. L. Davey, Frances Meeten, Andy P. Field

**Affiliations:** School of Psychology, The University of Sussex, Brighton, BN1 9QH UK

**Keywords:** Worrying, Anxiety, Students, Student Worry Questionnaire, PSWQ

## Abstract

**Background:**

The frequency and severity of mental health problems in student populations have been a growing cause for concern worldwide, and studies have identified measures of a number of mental health symptoms that have been steadily increasing in frequency and intensity over the past 20–25 years.

**Methods:**

In two studies we investigate the levels and domains of pathological worrying in university student participants. Study 1 is a retrospective study of Penn State Worry Questionnaire (PSWQ) data collected between 2001 and 2019. Study 2 describes the development of the Student Worry Questionnaire, a short and easily delivered measure of student worrying that identifies both frequency of worry as well as the student-relevant domains across which worrying occurs.

**Results:**

Study 1 revealed a steady increase in student worry scores of around 20% between 2001 and 2019, with a significant positive correlation between year of data collection and mean PSWQ score. The domain scores in Study 2 indicated that academic work was a significantly higher worry than any of the other domains, and worries about intimate relationships and ‘what people think of me’ were also worries that scored higher than either financial or health worries.

**Conclusions:**

The present studies indicate that pathological worrying can be added to the list of anxiety- and stress-related symptoms that have been shown to be on the increase in student populations in recent decades, and we discuss whether these increases represent a greater willingness to report symptoms or a genuine increase in experienced symptoms over time.

College and University students are regularly used as participants in psychological studies and, in particular, as analogue participants in mental health-related and psychopathology research. But students are a population experiencing very particular stress-related challenges from a variety of sources. These include the challenges of acute periods of intensive learning, living away from home for the first time and lacking access to key support networks, exposure to drink and drug-related activities, and student debt—and many of these factors have been shown to contribute to poorer psychological functioning (e.g. Brown, 2016; Cooke et al., 2004). As a result of these demands, longitudinal studies have indicated that student distress rises on entry to college and does not return to pre-college levels until the end of their course (Bewick et al., 2010), up to one in three students reports clinical levels of psychological distress (Bewick et al., 2008), and it appears to be an international phenomenon that affects students in many different countries (Rückert, 2015).

One way in which the psychological distress experienced by students is manifested is as chronic or pathological worrying and an increased risk for anxiety disorders such as Generalized Anxiety Disorder (GAD), of which pathological worrying is the cardinal diagnostic feature (Farrer et al., 2016; Pedrelli et al., 2015). While worrying is a cognitive activity that some people find helpful when it comes to problem solving and dealing with future threats and challenges, for many other individuals worrying can become a chronic and pathological activity. This pathological nature of worrying is characterized by a number of features—worrying begins to feel uncontrollable, a bout of worrying becomes perseverative and difficult to stop, and worrying increases rather than decreases levels of anxiety (Davey & Meeten, 2016; Davey & Wells, 2006). Worrying is an activity closely related to anxiety and stress, but has been shown to be a construct that is conceptually independent of anxiety and stress—an individual can worry without being anxious and can be anxious without worrying (Davey et al., 1992), and as a result worrying is worthy of research as a mental health problem in its own right, particularly if treatments and interventions for pathological worrying are to be successfully developed.

Another important feature of the stresses and anxieties experienced by college and university students is that the prevalence of these mental health symptoms is not static but is likely to change with the unique stressors experienced by students and also with changes in the nature and frequency of psychosocial stressors in society generally. The evidence suggests that the reporting of anxiety-related symptoms has increased significantly in recent years, both in young people generally and in student populations specifically. For example, in a longitudinal study of self-reported anxiety in Sweden, Calling et al. (2017) found an increasing prevalence of self-reported anxiety in young people (aged 16–23 years) in the 25 years between 1980 to 2005, and Pitchforth et al. (2018) found a ‘striking’ increase in the reporting of long-standing mental health conditions in young people (aged 4 to 24 years) between 1995 and 2014, and particularly in young adults since 2011. More focussed studies have shown that this increase in anxiety and mental health related conditions in recent times is not just a feature of young people generally, but can also be identified in specific populations such as college and university students. For instance, a survey of 38,000 university students in the UK found significantly high levels of anxiety across students in all 3 years of their undergraduate degrees, with 42.8% reporting being often or always worried (Pereira et al., 2019), resulting in a 1% increase in students needing professional mental health help for these conditions in just 1 year. Also, in a recent retrospective study of measures of ‘intolerance of uncertainty’, Carleton et al. (2019) found that levels of intolerance of uncertainty had been steadily increasing in college students between the years 1999 and 2014. Intolerance of uncertainty is a trait-like construct reflecting an inability to endure uncertainty, and has been shown to have a role in the development and maintenance of chronic and pathological worrying (Carleton, 2012; Freeston et al., 1994). In their study, Carleton et al. (2019) also found an upward trend in worry scores between 1999 and 2011 in studies that took this measure, but the slope of this increase failed to reach statistical significance.

Such findings have important implications for both the study and treatment of stress-related conditions in university and college students. First, there may be implications for the measurement of mental health conditions when using students as a research population. The steady increase in the reporting of stress-related symptoms by students over the past 20 to 25 years will require regular updating of norms for mental health instruments used with this population—especially if statistical deviations from norms calculated 20 years ago have been used as a way of identifying individuals currently at risk of being diagnosed with a specific mental health disorder. Secondly, a clear understanding of how and why reporting of mental health conditions in students has steadily changed over the years is necessary (1) to inform levels of mental health services for students (e.g. Broglia et al., 2018), and (2) to drive the development of treatment interventions and prevention programmes for student mental health problems.

In this respect, the aim of the present research is twofold: (1) to conduct a retrospective analysis of worry frequency scores reported by students at the University of Sussex over the past 20 years; this should provide information on whether there has been a steady increase in reports of worrying in the student population over this time; and (2) to develop a new instrument for measuring student worry—one that will provide information on both the frequency of worry in students and the domains of worry across which student worry occurs. Such an inventory should provide more detailed information on the causes of worrying in student populations, and provide data on the topics that generate student worrying and may need to be addressed by university welfare services.

## Study 1

For over two decades our research group at the University of Sussex has been conducting research on the causes of pathological worrying and as a result has collected 20-years’ worth of data on the frequency and severity of pathological worrying. Most of this research has been analogue research carried out on student participants, and the measure of pathological worrying used across these years has usually been the Penn State Worry Questionnaire (PSWQ) (Meyer et al., 1990).

In order to investigate whether levels of worrying in university students have been on the increase over the past 20 years we have re-analysed the PSWQ scores from 18 studies conducted with student participants between the years 2001 and 2019. The PSWQ is a self-report instrument for measuring the tendency for an individual to engage in excessive, generalized, and uncontrollable worry (Molina & Borkovec, 1994). It is a 16-item inventory derived from clinical and research experience with GAD patients and worriers, and represents a trait measure of the general tendency to worry without regard to content-specific topics. The PSWQ has good internal reliability in individuals with a diagnosis of GAD, community samples, and undergraduate students, with Cronbach’s alphas ranging from .88 to .95 (Startup & Erickson, 2006). It has demonstrated good test–retest reliability (Meyer et al., 1990), and high convergent validity with other worry measures (Davey, 1993). It also has high discriminant validity and correlates highly with measures of anxiety and trait anxiety (Meyer et al., 1990; Davey, 1993). Early studies on groups of nonanxious and anxious participants in the 1990s (selected using GAD-Q screening) indicated a group mean PSWQ score for nonanxious participants of 44.2 and for anxious participants meeting diagnostic criteria for GAD of 63.2. To our knowledge, the earliest mention of a potential PSWQ norm score for college student participants was by Startup and Erickson (2006) who noted a mean score of 47.4 in unselected college students, and a score of 42.6 in unselected community adult samples.

This first study traces possible changes in PSWQ scores across 18 studies identified at the University of Sussex and conducted on student participants between 2001 and 2019. All data used in this analysis were collected before the onset of the 2020 COVID-19 pandemic and the lockdowns associated with that pandemic.

### Method

#### Participants

Eighteen research studies were identified that had collected PSWQ scores and had been carried out between 2001 and 2019 with participants who were current undergraduate or taught postgraduate students at the University of Sussex. Of these 18 studies, 3 were questionnaire surveys and the remaining 15 were lab-based experimental studies. In all experimental studies PSWQ scores were collected at the outset of the experimental study and prior to any experimental manipulations taking place. The total number of participants in these studies was 1368, consisting of 392 males and 976 females. The mean age of participants across all studies was 24.4 years (SD = 8.1 years), with the mean age for males 26.5 years (SD = 10.1 years), and for females 23.5 (SD = 7.1). This age difference was statistically significant [*t*(1366) = 5.22, p < 0.001]. The number of participants in individual studies ranged from 40 to 217 with the mean number of participants per study being 76.

Table 1 provides details of participants in the 18 studies, including the years the studies were conducted, the N in each study, mean age of participants in each study, and the ratio of females to males in each study.

**Table 1:** Details of the 18 studies retrospectively analyzed in Study 1

	Year data collected	PSWQ (Mean + SD)	N	Mean age (years)	Male	Female	% Females
Study 1	2001	48.48 (13.30)	40	27.0	23	17	42.5
Study 2	2001	48.08 (13.29)	120	28.0	50	70	58.3
Study 3	2001	46.46 (11.49)	90	23.0	29	61	67.7
Study 4	2003	48.62 (10.19)	64	25.6	30	34	53.1
Study 5	2003	48.53 (10.61)	45	23.9	20	25	55.5
Study 6	2005	49.08 (9.16)	45	26.4	17	28	62.2
Study 7	2003	46.39 (9.89)	87	26.2	41	46	52.8
Study 8	2004–2005	51.48 (11.09)	60	25.2	11	49	81.6
Study 9	2007	47.32 (12.70)	124	22.2	17	107	86.2
Study 10	2006–2007	51.20 (14.13)	60	31.6	17	43	71.6
Study 11	2009–2010	50.69 (10.11)	62	25.6	16	46	74.1
Study 12	2010–2011	50.19 (13.45)	46	26.7	13	33	71.7
Study 13	2011–2012	55.73 (11.58)	60	25.2	24	36	60.0
Study 14	2012–2013	52.30 (11.70)	60	21.0	11	49	81.6
Study 15	2013–2014	56.58 (11.90)	60	21.8	5	55	91.6
Study 16	2014–2015	53.01 (12.00)	60	21.6	7	53	88.3
Study 17	2015–2016	52.03 (15.11)	69	30.9	19	50	72.4
Study 18	2019	57.39 (9.87)	216	20.1	42	174	80.5
Total			1368		392	976	

See text for further information

### Results

**Fig. 1:**
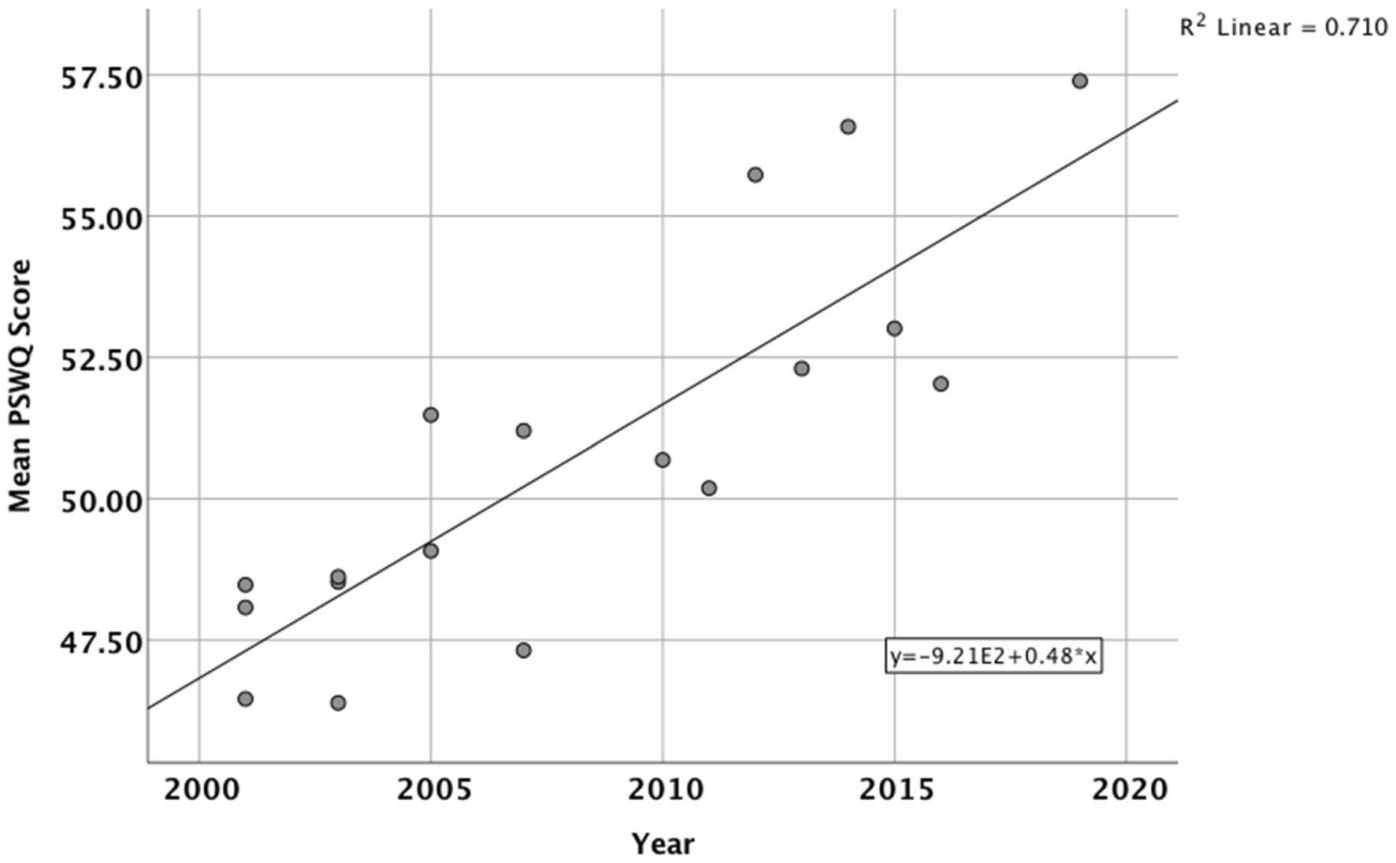
Scattergram and line of best fit showing the relationship between year of data collection and mean PSWQ score for all 18 studies analysed in Study 1

Table 1 also gives details of the mean PSWQ score (and SD) for each of the 18 studies. The correlation between the year of data collection and mean PSWQ score was R(18) = .86, p < .001, and this relationship between year of data collection and PSWQ is shown in Fig. 1. When mean PSWQ scores were analyzed in 5 year blocks based on the year that data collection started (2000–2004, 2005–2009, 2010–2014, 2015–2019), there was a significant effect of year block [*F*(3,14) = 7.82, *p* < .003], with pairwise comparisons indicating that PSWQ mean scores in the 2015–2019 block and the 2010–2014 block were significantly higher than those in the 2000–2004 block (both *p*s < .05, both *r*s > 0.74). Individual participant data was available for all studies with a data collection start date of 2003 or later (15 studies with a total N = 1118). Using these individual participant data, across all 15 studies, the mean PSWQ score for male participants was 47.60 (SD = 11.28) compared to 54.31 (SD = 11.54) for females. This sex difference in PSWQ scores was significant [*t*(1116) = 8.26, *p* < .001, *r* = 0.24]. Nevertheless, there was still a significant positive correlation between year of data collection and PSWQ scores for both males [*R*(290) = .12, p < .04] and females [*R*(828) = .26, *p* < .001].

**Fig. 2:**
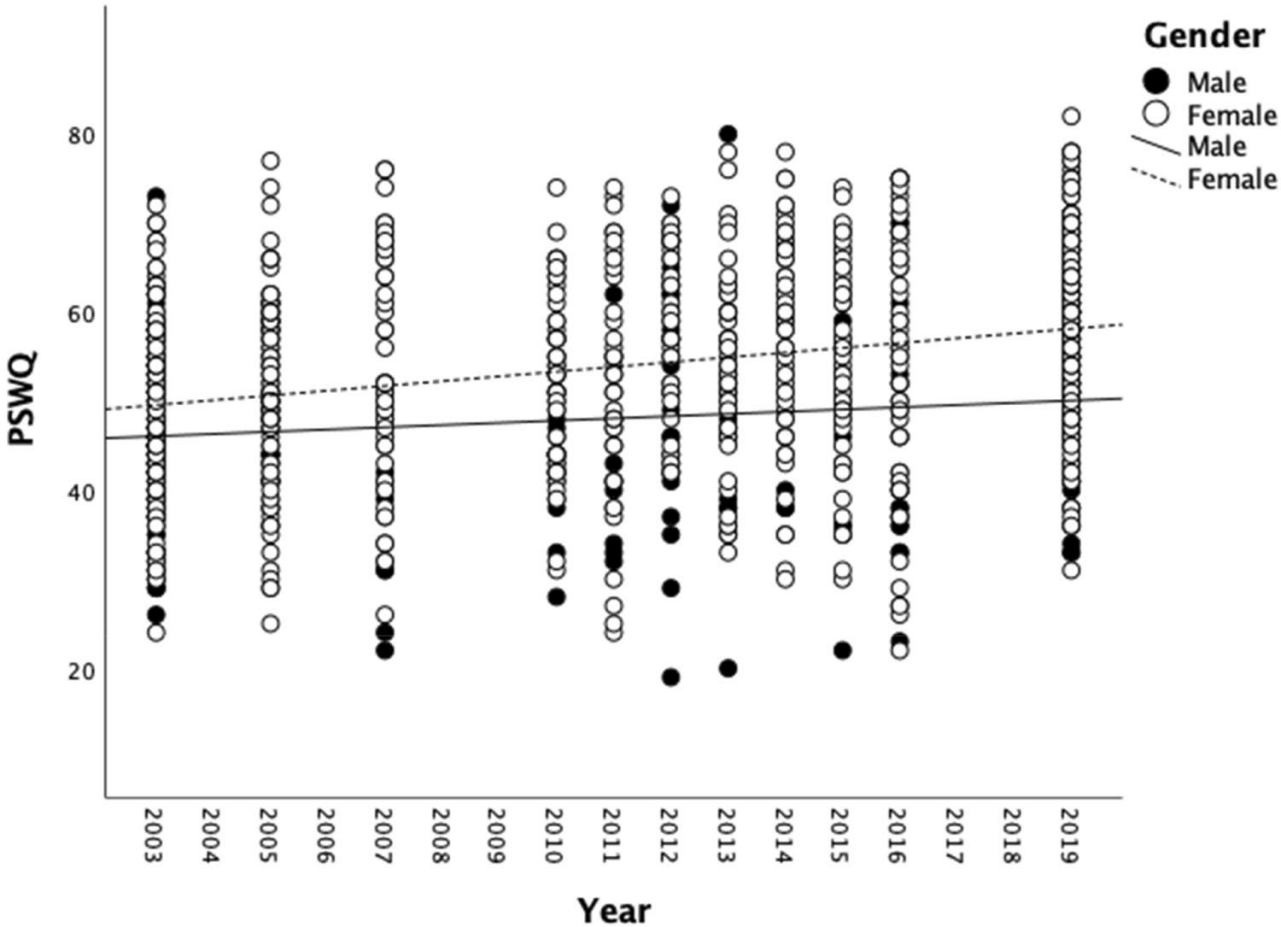
Individual PSWQ scores for males and females by year with lines of best fit

Potential confounding factors influencing the significant correlation between year of data collection and mean PSWQ score were possible differences in the mean age of the participants across different years and the proportion of males to females in each sample. There was no significant correlation between the mean age of participants and mean PSWQ score [*R*(18) =  − .30, *p* = .21), but there was a significant correlation between the percentage of females in each study sample and mean PSWQ score [*R*(18) = .53, *p* < .03]. To further examine this issue, we performed a bootstrapped regression analysis (with 1000 replications) on individual participants’ PSWQ scores to examine the relationship between year and PSWQ when also adjusting for gender, age and the gender × year interaction term in the model. Gender was entered into the model (as a known predictor of worry) in step 1, age in step 2, gender × year interaction term in step 3, and year in step 4. The model (when all predictors were entered into it) was a significant predictor of PSWQ scores *F*(4, 989) = 34.09, *p* ≤ .001. See Table 2 for the bootstrapped coefficients for step 4 of the model where we examine the effect of year on PSWQ when taking into account, gender, age, and the gender × age interaction term. When gender, age, and the gender × year interaction term are included in the model, then year remains a significant predictor, although gender and gender × year are also significant predictors. Looking at the PSWQ scores for males and females by year (Fig. 2), we can see that females tend to report higher PSWQ scores than males (as is often reported in the worry literature, e.g. Robichaud et al., 2003), and the line of best fit slopes suggest that increases in PSWQ across years was higher in females than males.

**Table 2:** Regression coefficients, significance values and 95% confidence intervals when gender, age, gender x year and year are included in the model

Step 4	*b*	SE	*p*	95% CI
Constant	− 385.67	213.01	.069	− 800.30, 18.55
Gender	5.76	0.85	< .001	4.17, 7.47
Age	− 0.09	0.05	.068	− 194, 0.01
Gender × year	1.71	0.73	.018	0.22, 3.15
Year	0.21	0.12	.040	0.01, 0.42

Note R^2^ for Step 4 = .12

### Discussion

This retrospective analysis of student PSWQ scores between 2001 and 2019 shows a steady increasing trend in these scores across two decades from 2001, with the best fit line in Fig. 1 suggesting an increase of around 20% from a mean PSWQ score of around 47 in 2001 to a mean score of 57 in 2020. There was a significant positive correlation between year of data collection and PSWQ score that remained significant even when controlling for Gender, although PSWQ scores overall were significantly higher in females and there was evidence suggesting that the rate of increase in PSWQ scores across years may have been higher in females than males. This gender difference is consistent with evidence in the literature that worry frequency is generally higher in females than males (McCann et al., 1991; Robichaud et al., 2003; Stavosky & Borkovec, 1987). This gradual increase in student worry scores over the past two decades is consistent with other reports of increases in anxiety and stress-related symptoms in student populations (Carleton et al., 2019; Pereira et al., 2019) and young adults generally (Calling et al., 2017; Pitchforth et al., 2018), and this increasing trend in PSWQ scores has also been reported in students in at least two other UK Higher Education institutions (Freeston, 2020; Morriss, 2020).

It is not easy to determine what has been causing these gradual increases in PSWQ scores over the last two decades. It could be a result of an increase in the number and frequency of psychosocial stressors affecting this age group in society generally (e.g. increased mobile phone penetration, and social media and internet usage, cf. Carleton et al., 2019; Davey, 2018), or it could be an effect of changes in higher education that specifically affect college and university students (e.g. the introduction of tuition fees in the UK, changes in teaching and assessment methods, and post-college job availability). Only studies comparing student populations with similar aged community samples will shed light on these alternatives, although existing community-based studies do suggest that stress and anxiety levels as well as the frequency of common mental health problems have been gradually increasing in young adults generally over the past 25 years (Calling et al., 2017; McManus et al., 2016; Pitchforth et al., 2018).

However, one plausible alternative explanation that deserves some investigation is that anxiety and stress levels per se may not have increased in recent years, only that the reporting of them has increased (see also discussion of this issue in the “General Discussion” section). This could be a result of a weakening of the taboos on reporting mental health symptoms or a growing awareness of mental health symptoms in young people as a result of modern-day mental health education.

Whatever the causes, this gradual rise in self-reported worry symptoms by students may have significant implications for student mental health—especially if these increases do reflect the effects of external factors that drive stress and anxiety. Higher PSWQ scores imply an increase in worrying being perceived as chronic and uncontrollable, and perseverative worrying has been shown to be caused by high levels of negative mood (Meeten & Davey, 2011), with levels of stress and anxiety increasing as a worry bout proceeds (Davey et al., 2007). As well as these increases in stress and anxiety associated with chronic worrying, the highest PSWQ scores are usually associated with individuals receiving a diagnosis of GAD, which is to be expected because the cardinal diagnostic criterion of this condition is uncontrollable chronic worrying (APA, 2013). Startup and Erickson (2006) reported a mean PSWQ score of 67.16 derived from studies of clinical samples of adults with a diagnosis of GAD which is 10 points above the most recent means reported for our student populations. However, we should be cautious about whether high scores on the PSWQ are indicative of an anxiety disorder such as GAD. Whereas high scores on the PSWQ may represent high levels of worrying that reflect underlying stress and anxiety, they do not necessarily indicate a diagnosis of GAD. For example, although non-GAD high worriers commonly regard their worry as uncontrollable, they perceive more control over their worry than do GAD high worriers (Ruscio, 2002), and a diagnosis of GAD implies more than just high levels of uncontrollable worrying, but also a pattern of anxious, somatic and depressive symptoms (Slazer et al., 2009). So, while high PSWQ scores may indicate that an individual may be at risk for developing GAD, it is not an entirely reliable instrument to use in screening for GAD.

While the PSWQ is a reliable measure of the frequency and severity of pathological worrying, it does not convey information about the content of worrying. Given that the self-reported severity of student worrying has increased significantly over the past 20 years, it would be beneficial to begin to understand the domains around which worrying occurs in this population. This should enable us to begin to understand the causes of student worry and to target these causes with preventative and ameliorative programmes. In this respect Study 2 describes the development of a short questionnaire to measure the frequency and content of student worry.

## Study 2

Both non-pathological and pathological worriers report that a significant majority of their worrying is in response to recognisable precipitants that represent events and challenges in their own lives (Craske et al., 1989), and so identifying threats and challenges relevant to different individuals can be helpful when trying to find ways to help them manage chronic worrying.

In this respect, the aim of Study 2 was to develop a measure of student worrying (the Student Worry Questionnaire, SWQ) that captured both the intensity and frequency of student worry as well as the frequency of worrying across a number of domains relevant to the lives of a student population. The frequency measure would help to identify students for whom worrying had become a chronic and distressing activity, and the domains measure would help to identify the primary sources that were precipitating worrisome thinking.

### Method

#### Participants and Procedure

All participants were undergraduate or post-graduate psychology students at the University of Sussex. Participants completed the questionnaire on line in return for a course credit. For the Exploratory Factor Analysis (Study 2a), data were collected between March 13th and May 10th 2019, and only the last 2 weeks of this period fell during the time students would be taking exams and year-end assessments. There were 216 participants, 42 males and 174 females. Overall average age was 20.1 years (SD = 3.44), with a range of 18 to 46 years. Males mean age was 20.5 years (SD = 3.14) and females mean age was 20.0 years (SD = 3.52), this age difference was nonsignificant [*t*(214) = .84, p > .40, ns]. For the Confirmatory Factor Analysis (Study 2b), data were collected between November 8th 2019 and December 12th 2019. There were 197 participants, 32 males, 163 females and 1 unspecified. Overall average age was 19.7 years (SD = 3.87), with a range of 17 to 70 years. Males mean age was 21.6 years (SD = 9.05) and females mean age was 19.3 (SD = 1.42), this age difference was nonsignificant [*t*(192) = 1.44, p = .15, *ns*]. Participants in Study 2a did not contribute to Study 2b. The study was approved by the University of Sussex Sciences & Technology Ethics Committee.

#### Questionnaires

##### Development of the Student Worry Questionnaire (SWQ)

To generate items for the SWQ a student focus group was convened consisting of 11 students all aged 18 years, and with an equal representation of males and females. The group was asked to discuss the kinds of things they worried about and to finish this discussion by agreeing a list of six worry domains that they considered were the most common topics of worrying for students. These six items would form the domains sub-scale of the SWQ. The six items for the frequency sub-scale of the SWQ were selected from a pool of items adapted from the Penn State Worry Questionnaire (Meyer et al., 1990) and the Worry Stop Rules Questionnaire (Davey et al., 2005) to represent questions addressing the frequency and intensity of worrying. The 12-items selected to make up this initial version of the SWQ are listed in Table 3. For each item a rating on a four-point scale is required (1 = never true of me, 2 = sometimes true of me, 3 = most times true of me, 4 = always true of me).

**Table 3:** Items making up the Student Worry Questionnaire (SWQ) used in Study 2

This questionnaire is about worrying. Worrying is when you’re stressed about something and you think about it a lot. Read each sentence below and then circle the answer you think best describes how true that sentence is about you *during the past week*				
1. I can’t stop worrying until I’ve solved everything about my worry (F)	Never true	Sometimes true	Most times true	Always true
2. I must think about the worst possible outcome of my worry, just in case it happens (F)	Never true	Sometimes true	Most times true	Always true
3. I just can’t sit back and forget about my worries (F)	Never true	Sometimes true	Most times true	Always true
4. I worry a lot (F)	Never true	Sometimes true	Most times true	Always true
5. My worries make me feel stressed (F)	Never true	Sometimes true	Most times true	Always true
6. I find it difficult to stop worrying (F)	Never true	Sometimes true	Most times true	Always true
7. I find it difficult to stop worrying about my academic work, such as exams and deadlines (D)	Never true	Sometimes true	Most times true	Always true
8. I find it difficult to stop worrying about my health (D)	Never true	Sometimes true	Most times true	Always true
9. I find it difficult to stop worrying about my financial circumstances (D)	Never true	Sometimes true	Most times true	Always true
10. I find it difficult to stop worrying about my intimate relationships (D)	Never true	Sometimes true	Most times true	Always true
11. I find it difficult to stop worrying about what others think of me (D)	Never true	Sometimes true	Most times true	Always true
12. I find it difficult to stop worrying about family issues (D)	Never true	Sometimes true	Most times true	Always true

*F* frequency item, *D* domain item

##### The Penn State Worry Questionnaire (PSWQ)

The Penn State Worry Questionnaire (Meyer et al., 1990) is a valid measure of trait worrying that is unaffected by the content of the worry (Molina & Borkovec, 1994; Davey, 1993). The PSWQ is a 16-item one-page measure of trait worrying. For each statement a rating on a five-point scale is required (1 = not at all typical of me, 2 = rarely typical of me, 3 = sometimes typical of me, 4 = often typical of me, 5 = very typical of me). The PSWQ has good internal reliability in individuals with a diagnosis of GAD, community samples, and undergraduate students, with Cronbach’s alphas ranging from .88 to .95 (Startup & Erickson, 2006).

At the end of the questionnaire, participants in Study 2a were also asked three short questions about their use of smartphone apps to help them manage their mental health. These questions were not relevant to the present study.

### Results

For the factor analyses sections of the results, we used R version 4.1.0 (R Core Team, 2021) for all analyses, the lavaan (Rosseel, 2012) package and the tidyverse suite (Wickham et al., 2019). Items on the SWQ used Likert scales and so models were fit using the polychoric correlation matrix of items (Field, 2022; Wirth & Edwards, 2007).

#### Exploratory Factor Analysis

The KMO test yielded overall sampling adequacy in the Marvellous category, 0.90 and for individual items it ranged from 0.83 to 0.95, which are all in the meritous and marvellous category (Kaiser & Rice, 1974). Parallel analysis (Horn, 1965) based on factor analysis suggested 3 underlying factors. Consequently, a factor analysis with oblique rotation (oblimin) was fitted using weighted least squares estimation (WLSMV) on the polychoric correlations between items. Table 4 shows the fit statistics. Good fit is indicated by a combination of TLI > 0.96 and SRMR < 0.06 and a combined rule of RMSEA < 0.05 and SRMR < 0.09 (Field, 2022). The current model has adequate fit.

**Table 4:** Fit indices for exploratory models

Model	${\chi }^{2}$	df	$p$	RMSEA [90% CI]	TLI	SRMS
Three factor (EFA)	45.68	33	.070	0.04 [0.00, 0.07]	0.99	0.03
Bi-factor (3 group)	58.38	39	.024	0.05 [0.02, 0.07]	0.99	0.04
Three factor (CFA)	151.57	51	< .001	0.10 [0.08, 0.12]	0.92	0.08

Table 5 shows the factor loadings after rotation, and seems to indicate that factor 1 might be general worry (items 1 to 6 load highly onto this factor). As would be expected from the questions, Factors 2 and 3 relate to specific worries. Items 7 to 9 load highly onto factor 2 and relate to non-social worries, factor 3 has items relating to social worries (items 10 to 12), however item 12 loaded equally on factors 2 and 3, and item 11 loaded equally onto factor 1 and 3. Factor 1 correlated very highly with factor 2 (0.65) and factor 3 (0.47) and factors 2 and 3 also correlated strongly (0.34).

**Table 5:** Factor loadings for the SWQ items on 3-factors

rhs	Factor 1	Factor 2	Factor 3
Item 01	0.78	0.04	− 0.15
Item 02	0.70	− 0.02	0.11
Item 03	0.90	− 0.10	− 0.06
Item 04	0.73	0.13	0.13
Item 05	0.60	0.29	0.03
Item 06	0.69	0.09	0.21
Item 07	0.14	0.72	− 0.07
Item 08	− 0.09	0.52	0.13
Item 09	− 0.03	0.61	0.02
Item 10	0.03	0.00	0.83
Item 11	0.37	− 0.02	0.38
Item 12	0.03	0.32	0.32

#### Bifactor Model

Given the strong correlation between factors and the fact that items 1 to 6 (the items relating to general worry) clustered together, it is possible that a bi-factor model is a more appropriate way to conceptualise the questionnaire (Lorenzo-Seva & Ferrando, 2019): that is, there is a general superordinate factor that sits above the three identified factors. Such a model was fitted, however, the fit indices were slightly worse (Table 4) suggesting that we could stay with the three factor model.

#### Confirmatory Factor Analysis

A CFA was performed on sample 2 forcing the three-factor structure that emerged from the exploratory factor analysis. That is, three correlated latent variables were defined: general worry (indicated by items 1 to 6), nonsocial worries (items 7 to 9), and social worries (items 10 to 12). Table 4 shows that this model was a poor fit of the data. The general worry factor correlated very highly with the nonsocial worry factor (0.84) and the social worry factor (0.87) and the nonsocial and social worry factors also correlated strongly (0.87). Table 6 shows the standardized parameter estimates for each item and its corresponding factor.

**Table 6:** Standardized parameter estimates for SWQ items on 3-factors in a CFA

Factor	Item	$\beta$	Lower	Upper	$z$	$p$
General	Item 01	0.66	0.56	0.75	13.74	0.00
General	Item 02	0.78	0.70	0.85	19.92	0.00
General	Item 03	0.65	0.55	0.74	13.69	0.00
General	Item 04	0.86	0.79	0.92	25.07	0.00
General	Item 05	0.79	0.72	0.86	21.43	0.00
General	Item 06	0.88	0.82	0.93	32.84	0.00
Nonsocial	Item 07	0.78	0.65	0.92	11.40	0.00
Nonsocial	Item 08	0.48	0.35	0.62	6.86	0.00
Nonsocial	Item 09	0.41	0.27	0.54	5.83	0.00
Social	Item 10	0.40	0.26	0.54	5.69	0.00
Social	Item 11	0.61	0.47	0.76	8.45	0.00
Social	Item 12	0.45	0.31	0.59	6.22	0.00

When data from Studies 2a and 2b were combined, Cronbach’s alpha for the 6-item General Worry Scale (Q1–Q6) was 0.86, and for the six domain items was 0.65. While scale reliability scores for the General Worry Scale and full domains scales were acceptable to good, Cronbach’s alpha for the two domains sub-scales were less than moderately acceptable at 0.54 for the three non-social worry items and 0.53 for the three social worry items. However, it is worth noting that the size of alpha depends on the number of items so low values for the subscales with three items need to be interpreted in this context.

Table 7 shows the main descriptive statistics for the SWQ, its factor sub-scales and the PSWQ. The main point of note is that females scored higher than males on all measures in Study 2a, but only on the PSWQ measure in Study 2b.

**Table 7:** Mean scores + standard deviations for the total SWQ score, the 6-item SWQ General Worry Scale, and the Penn State Worry Questionnaire (PSWQ)

	All participants (Study 2a)	Male (Study 2a)	Female (Study 2a)	All participants (Study 2b)	Male (Study 2b)	Female (Study 2b)
SWQ total	32.30 (6.83)	27.02 (7.18)	33.58 (6.06)*	32.56 (6.33)	30.65 (7.06)	32.94 (6.15)
SWQ (General Worry Scale)	17.13 (4.18)	13.68 (3.90)	18.00 (3.79)*	17.52 (3.66)	16.43 (4.36)	17.72 (3.74)
PSWQ	57.12 (10.09)	50.97 (9.25)	58.65 (9.71)*	58.55 (10.88)	54.67 (11.44)	59.36 (10.70)*

*Scores significantly higher for females than males (all *t*s > 2.11, all *p*s < .05)

**Fig. 3:**
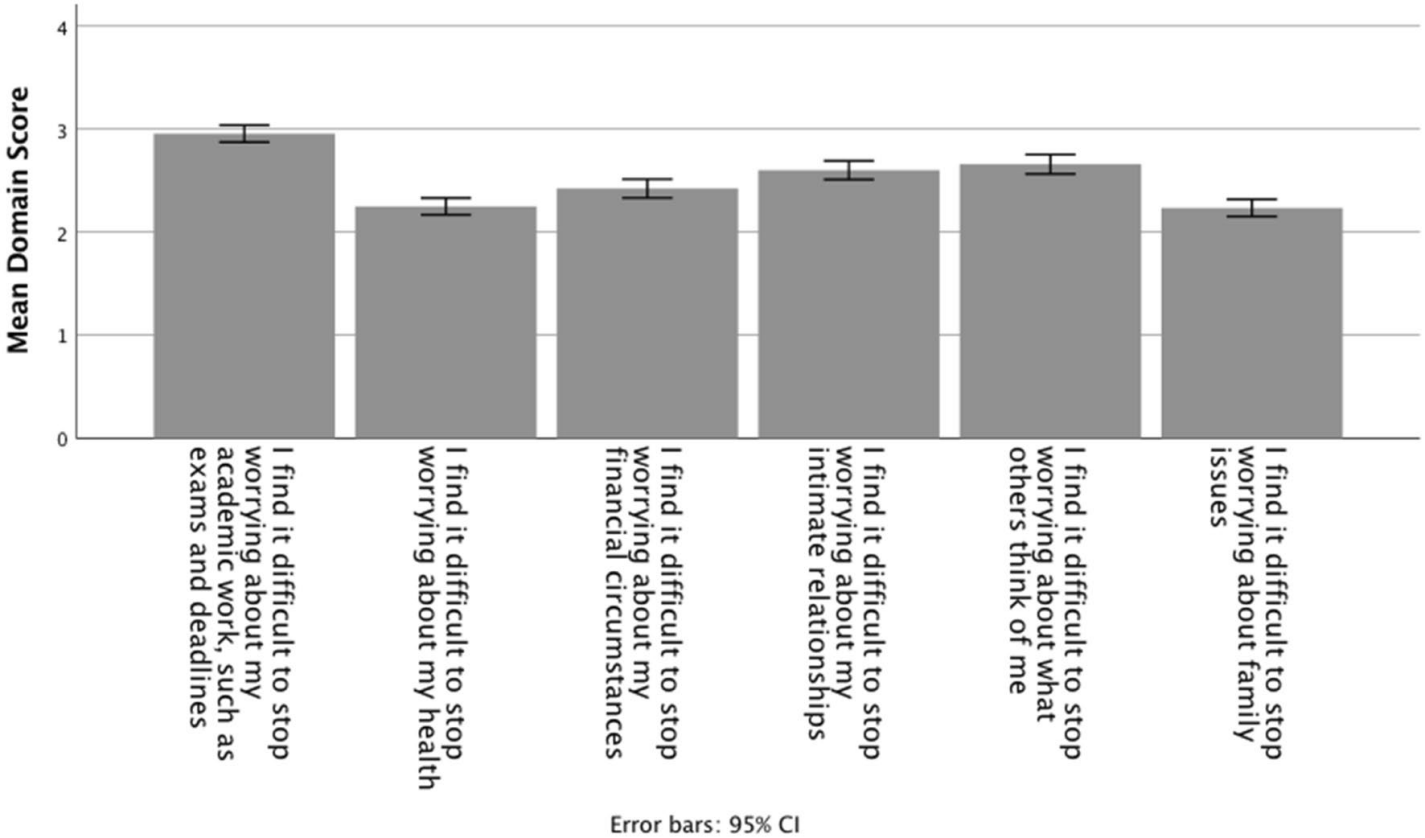
The mean scores for each of the domain-specific items of the SWQ (items Q7–Q12, combined data from Studies 2a and 2b)

Because reliability of the two domain sub-scales was relatively low, it was considered more instructive to compare information across the individual worry domain items to identify topics that are contributing to student worry. Figure 3 shows the mean scores for each of the domain-specific items of the SWQ (combined data from Studies 2a and 2b). As we might expect, pairwise comparisons indicate that the mean score for worrying about academic work (Q7) is significantly higher than the scores for worrying in any of the other five domains (Q8–Q12) (all *t*s > 5.37, all *p*s < .001, all *r*s > .38). In addition, worry about intimate relationships (Q10) and ‘what people think of me’ (Q11) both score higher than both worry about finances (Q9) and worry about health (Q8) (all *t*s > 3.10, all *p*s < .002, all *r*s > .09), suggesting that worries about the financial pressures of studying for a degree may be less demanding than the burdens of developing and maintaining relationships.

Table 8 shows correlations between the PSWQ, the 6-item General Worry Scale and individual domain items of the SWQ. This indicates that the SWQ General Worry Scale scores are highly correlated with scores on the PSWQ, testifying to the construct validity of the SWQ General Worry Scale. In addition, all individual domain items of the SWQ were highly correlated with both the SWQ frequency scale score and scores on the PSWQ.

**Table 8:** Correlations between the PSWQ, the SWQ General Worry Scale, and individual domain items of the SWQ

	PSWQ	SWQ (General Worry Scale)	Q7	Q8	Q9	Q10	Q11	Q12
PSWQ								
SWQ (General Worry Scale)	.81**							
Domains Q7 (academic)	.54**	.54**						
Domains Q8 (health)	.32**	.28**	.26**					
Domains Q9 (finances)	.30**	.28**	.34**	.25**				
Domains Q10 (relationships)	.26**	.32**	.15**	.22**	.19**			
Domains Q11 (what others think)	.46**	.47**	.25**	.17**	.08	.33**		
Domains Q12 (family)	.29**	.32**	.26*	.19**	.34**	.26**	.21**	

**p < .001

**Fig. 4:**
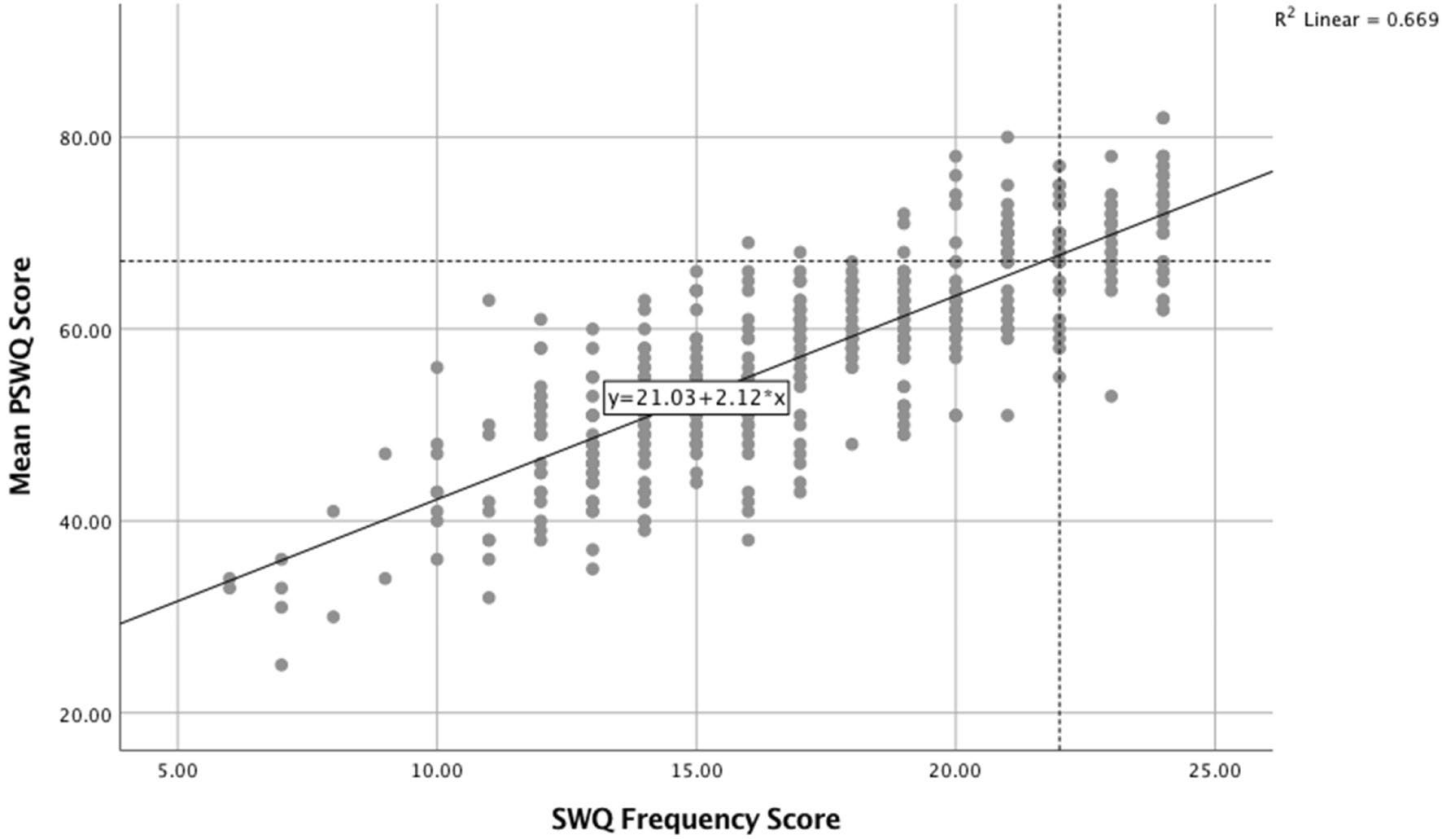
Scattergram and line of best fit showing the relationship between PSWQ scores and scores on the SWQ 6-item General Worry Scale. The horizontal and vertical dotted lines adjacent to the line of best fit show that a PSWQ score of 67.1, which was identified by Startup and Erickson (2006) as a mean PSWQ score for clinical samples with GAD, is equivalent to an SWQ General Worry Scale score of 22.0 (combined data from Studies 2a and 2b)

Finally Fig. 4 provides a scattergram with line of best fit comparing scores on the PSWQ with those from the SWQ General Worry Scale (items 1–6). The horizontal and vertical dotted lines adjacent to the line of best fit show that a PSWQ score of 67.1, which was identified by Startup and Erickson (2006) as a mean PSWQ score for clinical samples with GAD, is equivalent to an SWQ General Worry Scale score of 22.0. Students reporting scores above 22 on the SWQ General Worry Scale could therefore be considered as potentially at risk for GAD as a result of their levels of worrying.

### Discussion

The SWQ represents a short and easily delivered measure of student worrying that identifies both frequency of worry as well as the student-relevant domains across which worrying occurs. The six item SWQ General Worry Scale has good internal reliability and good construct validity. And the six items measuring worry across student-relevant domains all independently correlate highly with the SWQ General Worry Scale and an established measure of pathological worry such as the PSWQ.

In the cohort used to develop the SWQ, the domains scores indicated that academic work was a significantly higher worry than any of the other domains. This is obviously a worry domain that is at the heart of the student experience, and excessive worry about academic work does have negative consequences, including poorer academic outcomes associated with academic and assessment stress (McIntyre et al., 2018). Lipson and Eisenberg (2017) also identified worrying about coping with academic demands and worry about whether they would finish the course as significant predictors of mental health problems in students. Macaskill (2018) reported similar findings in which distressed students worried much more about their course work and student experience generally than did “well” students.

Worries about intimate relationships and ‘what people think of me’ were also important worry domains for our student cohort, and these worries reflect sources of stress that are important to all young people learning to navigate social interactions and, in particular, to the 16–19 year age group who may be entering more intense committed, relationships for the first time (Connolly et al., 2000; Furman & Shomaker, 2007). Interestingly, academic and relationship worries scored significantly higher than financial issues, suggesting that financial issues generally and financial difficulty resulting from increased tuition fees specifically may, in relative terms, be less important worries for students than has previously been assumed (e.g. Gani, 2017).

One limitation of this study is that the development of the SWQ was carried out only on students studying for a degree in psychology and only in one particular higher education institution, so there is still evidence required to demonstrate that the SWQ will be similarly applicable across students studying different disciplines, from different backgrounds, and studying in different educational institutions and settings. However, while we might expect to find discrete differences in worry intensity and domains as a function of degree discipline in particular, the overall picture in higher education is one in which there is growing public concern about student mental health across the whole sector, with student counselling services in both the UK and USA reporting significant increases in student helpseeking and the severity of student mental health issues (Avotney, 2014; Brown, 2018; Flatt, 2013).

## General Discussion

The frequency and severity of mental health problems in student populations have been a growing cause for concern worldwide. Studies that have longitudinal data on these mental health problems have identified measures of a number of mental health symptoms that have been steadily increasing in frequency and intensity over the past 20–25 years, and these include anxiety, psychological distress, self-harm, depression and suicidal ideation (Garlow et al., 2008; Ibrahim et al., 2013; Pereira et al., 2019). Measures of mental health-related cognitive constructs such as intolerance of uncertainty have also been found to have increased over time in the last 20 years (Carleton et al., 2019). Measures of pathological worrying can now be added to this list, with Study 1 describing a steady increase in PSWQ scores across the years from 2000 to 2019.

What is interesting about these increases in measures of mental health conditions in students is that the increases appear to have happened slowly and steadily and do not seem to be easily associated with a single identifiable cause. Researchers have pinpointed a number of factors that may be relevant to this rise in reported symptoms, and these include increased student numbers, increased class sizes, less personalized tutor support for students (Bathmaker, 2003), and increasing tuition fees (Gani, 2017). However, the increase in the reporting of mental health problems in student populations may simply be reducible to general psychosocial factors that have caused an increase in the reporting of mental health problems in young people generally (e.g. Calling et al., 2017; Pitchforth et al., 2018), and such factors might include the growing negative influence of social media and cyberbullying on young adults (Carleton et al., 2019; Davey, 2018; Jacobsen & Forste, 2011; Mishna et al., 2018), economic downturns in the early and mid-2000s, and a later entrance into the labour market for young adults as a result of increased educational demands in many countries (Calling et al., 2017). Further detailed research will be necessary to evaluate the relevance of each of these factors.

However, an alternative explanation for these increases in reporting of mental health problems in students and young people is not that symptoms per se have been increasing, but the *reporting* of symptoms has increased over time. For example, although they identified a striking increase in the reporting of mental health conditions in young people between 1995 and 2014, Pitchforth et al. (2018) surprisingly found little change in scores on questionnaires related to psychological distress and emotional well-being. Calling et al. (2017) noted that over time people have become more informed about mental health problems, and are more willing to discuss these problems. This would particularly be the case if levels of stigma associated with mental health problems have decreased over recent years. However, whether this is an explanation for the increasing levels of self-reported worrying found in Study 1 is unclear. It would require a retrospective comparison of self-reported worry measures with corresponding objective and independent measures of symptom frequency and severity to resolve this—and it is likely to be very hard to find studies that have such comparable retrospective epidemiological data available using the same methodological setting (Bandelow & Michaelis, 2015). An alternative that might shed some light on these possible explanations would be a retrospective comparison of implicit measures of worrying, such as attentional and interpretation biases (Hirsch & Mathews, 2012) taken over time, as well as behavioural measures of worrying such as the number of catastrophizing steps emitted by students in lab-based studies (Davey, 2006). Such measures should be less affected by the conscious awareness of mental health problems that might affect self-report measures of symptoms. However, even if the increase in student worry levels over the years is only a result of facilitated reporting, it is still likely to result in an increased demand for mental health and well-being services—a challenge that student counselling services are already aware of (Broglia et al., 2018).

The purpose of Study 2 was to develop a short measure of worrying in students that would capture both the frequency of worrying and the domains across which worrying occurs. In the present cohort the domains scale of the SWQ identified academic issues and relationships as the two important worry topics for students, both more important than finances and health. It is not surprising that academic issues come top of the list, and excessive worry about academic concerns is a predictor of mental health problems and poorer academic outcomes (Lipson & Eisenberg, 2017; Macaskill, 2018; McIntyre et al., 2018). Relationship issues are also a source of major concern for students who are at an age when they may be forging their first intense, committed relationships, and especially when seeking new friendships after leaving home and starting at university.

There is a clearly established relationship between excessive worrying in students and a significant risk of mental health problems (e.g. Lipson & Eisenberg, 2017), but it is still not clear in which direction this relationship runs. Prospective studies measuring worry and mental health symptoms will clarify this. However, rather than being a cause or effect of mental health problems, excessive worrying can itself be considered a significant element in the clusters of symptoms that define anxiety disorders such as GAD and obsessive–compulsive disorder (OCD) (Comer et al., 2004; Starcevic et al., 2007).

The main limitation of the present studies is that they are based on data from just one higher education institution and student participants studying only one particular discipline. In Study 1, it would have been helpful to also have other demographic information as well as a consistent measure of self-reported anxiety collected across the studies, but unfortunately these were not collected consistently enough across the studies to be included in the analyses. In Study 2, it would have aided construct and discriminant validity to include a range of self-reported anxiety measures to compare with the SWQ measure. This should be an important inclusion in future studies examining the SWQ. However, informal correspondence with researchers from two other UK HE institutions has confirmed the longitudinal increase in student worry scores in their own institutions over the last decade and a half (Freeston, 2020; Morriss, 2020), and future studies could focus on meta-analyses of PSWQ studies across the preceding decades in order to confirm the findings of Study 1. Finally, the present findings are also consistent with other broader ranging studies that have identified increases in self-reported anxiety and stress related symptoms in undergraduate students and will add pathological worrying to this growing literature (Carleton et al., 2019).
